# Clinical research stakeholders’ experiences of clinical research during COVID-19: a qualitative study

**DOI:** 10.1186/s13104-023-06534-5

**Published:** 2023-09-30

**Authors:** Christine FitzGerald, Aoife Vaughan-Witts, Louise Barry, Gillian Corey, Fiona Leahy, Siobhán Egan, Elaine Conway, Margaret O’Connor, Rose Galvin

**Affiliations:** 1 School of Allied Health, Faculty of Education and Health Sciences, Ageing Research Centre, Health Research Institute, University of Limerick, Limerick, Ireland; 2 School of Medicine, Faculty of Education and Health Sciences, University of Limerick, Limerick, Ireland; 3 Clinical Research Support Unit, University Hospital Limerick, Dooradoyle, Limerick Ireland; 4 Department of Ageing and Therapeutics, University Hospital Limerick, Dooradoyle, Limerick Ireland

**Keywords:** Clinical research, COVID-19, Clinical research stakeholders, Qualitative

## Abstract

**Background:**

The COVID-19 pandemic created a complex high-risk clinical research environment with clinical research activities significantly impacted. Clinical research stakeholders adapted rapidly to new clinical practices; PPE, infection control policies, all while engaging with a more unwell patient demographic. The aim of this study is to explore the experiences of conducting clinical research during COVID-19 with clinical research stakeholders.

**Methods:**

This qualitative study of semi-structured interviews conducted with clinical research stakeholders in an acute Hospital setting across a variety of disciplines; Consultant Geriatrician, Clinical Research Nurse, Occupational Therapy, Physiotherapy. Interviews were fully transcribed prior to reflexive thematic analysis. NVivo software was used to support data management and analysis.

**Results:**

Three main themes were produced; (1) The challenging COVID-19 clinical research landscape, (2) COVID-19 clinical research communication barriers, and (3) Adaptations and learnings from clinical research during COVID-19.

**Conclusions:**

This study explored the experiences of conducting clinical research during COVID-19 with clinical research stakeholders examining challenges faced and adaptations required. The findings inform, equip and support clinical research stakeholders in the event of future adverse public health events.

**Supplementary Information:**

The online version contains supplementary material available at 10.1186/s13104-023-06534-5.

## Background

COVID-19 has had a significant impact on healthcare and clinical research, causing significant disruption to the delivery of health services and suspension of clinical research activities [1]. Clinical research stakeholders (CRS) were doubly impacted, tasked with navigating challenges across clinical and academic settings. Impact on clinical research varied based on the stage of projects, ranging from recruitment disruptions, pausing data collection pausing and cessation [2]. Long-term effects continue to be felt across the clinical research landscape with ongoing patient concerns of COVID-19 exposure creating reluctance for clinical research participants [1, 2].

Existing research examining clinical research during COVID-19 focused predominantly on clinical research trials and biomedical research, [3–7], with limited Health and Social Care Professional (HSCP) consultations with CRS. A notable systematic review examining health care workers experiences of COVID-19 provided important insights into some HSCP groups, albeit with the absence of engagement with CRS, illustrating a gap in existing research [8]. While profiling of the broader COVID-19 landscape from the perspective of HSCPs is underway [9–11], specific experiences of CRS remain largely omitted, particularly studies capturing both perspectives of academics and clinicians involved in clinical research, with challenges and experiences of these groups during the pandemic largely unknown [8–15]. Therefore, this study aims to address this gap in the literature through exploring the experiences of conducting clinical research during COVID-19 with CRS in an acute Hospital in the Mid-West of Ireland. Anticipated outcomes of the study may include themes of personal and professional COVID-19 impacts, reutilisation of health systems and sources of support for CRS during COVID-19.

## Methods

### Study design and setting

This qualitative study of semi-structured interviews, utilising the COREQ standardised reporting guidelines to standardise conduct and reporting of the study [16]. The setting of the study is an acute University Hospital site in the Mid-West of Ireland, with specialist research units affiliated with the Hospital site. All clinical research activities relevant to this study were conducted in the Hospital site. The University Hospital is one of seven Hospital Groups in Ireland located in an urban setting.

### Participants

For the purpose of this study, a CRS was defined as: *an individual who was employed in the clinical or academic setting, involved in the following areas of clinical research; research design and coordination, recruitment, data collection, data analysis, research lead and research collaborator*. Participants were employed in a variety of clinical and academic positions (e.g., Consultant Geriatrician), as well as roles with the sole focus of clinical research (e.g., Clinical Research Nurse). Table 1 provides CRS roles, including Clinical Research Nurse, Consultant Geriatrician, Physiotherapist, Social Worker, Clinical Research Coordinator.

**Table 1:** Participant Demographic

Participant	Gender	Role
1	F	Clinical Research Nurse
2	F	Dietitian
3	F	Course Director in Clinical Nutrition & Dietetics
4	F	Consultant Geriatrician
5	M	Prof of Clinical Nutrition
6	F	Clinical Research Nurse
7	F	Clinical Research Nurse
8	F	Clinical Research Nurse
9	F	Clinical Research Nurse
10	F	Clinical Research Nurse
11	F	Clinical Researcher and CNM
12	F	Clinical Research Coordinator & Lecturer
13	F	Physiotherapist
14	F	Clinical Research Nurse
15	F	Physiotherapist
16	F	Pharmacist
17	F	Social Worker
18	F	Occupational Therapist

Participants were engaged in a range of clinical research activities during COVID-19, including experimental research, observational and cohort studies. Clinical research activities referred to in this study were at various stages; ranging from project set up, patient recruitment, intervention delivery, patient follow-up, and dissemination.

The study utilised a purposive sampling approach, with additional snowball sampling. The total sample size for the study was 18. Participants were deemed to meet inclusion criteria if they were actively involved in preparing, conducting, or disseminating clinical research during COVID-19 in the clinical or academic setting (between March 2020 – August 2021. Participants who were not involved in preparing, conducting, or disseminating clinical research between March 2020 – August 2021 were deemed not to meet the inclusion criteria.

Potential participants were identified by a key stakeholder with extensive experience as a Clinical Research Nurse (LB) at the University Hospital site. Participants were identified based on a register of active clinical research stakeholders and advised to contact the research team (AVW) who offered an invitation to take part in the study by email with a follow up phone call on the study overview, allowing for any questions to be raised. Participants interested in participating were sent a study information sheet and consent form to review.

### Data collection

Semi-structured interviews were conducted with eighteen CRS (n = 18) between June and August of 2021 (Wave 4 of COVID-19). Due to COVID-19 limitations, a combination of in person and phone interviews were conducted in line with public health measures. Participants had the option of taking part in the interview either in person or by phone. Written informed consent was obtained prior to participants taking part in the study.

The research team consisted of two female researchers (AVW and LB) with previous experience in conducting qualitative research. The research team was not involved in patient care, nor involved in previous research or clinical experiences with participants. The role of the research team was outlined to the participants, as well as the topic of the study.

The question guide for the semi-structured interviews was developed using an evidence-based framework to model question composition [17]. This framework was selected following a review of the literature and deemed to reflect the research question. Content and subject matter components of questions were derived from clinical and academic experiences amongst the research team during COVID-19 and following exploratory literature searches to address this research gap. Topics in the question guide explored key areas including clinical research of COVID-19 barriers and facilitators, support, and information resources. The question guide was piloted prior to use with stakeholders involved in clinical and academic health services research, with revisions made to ensure clarity on maintaining open-ended question structure.

### Data analysis

**Fig. 1:**
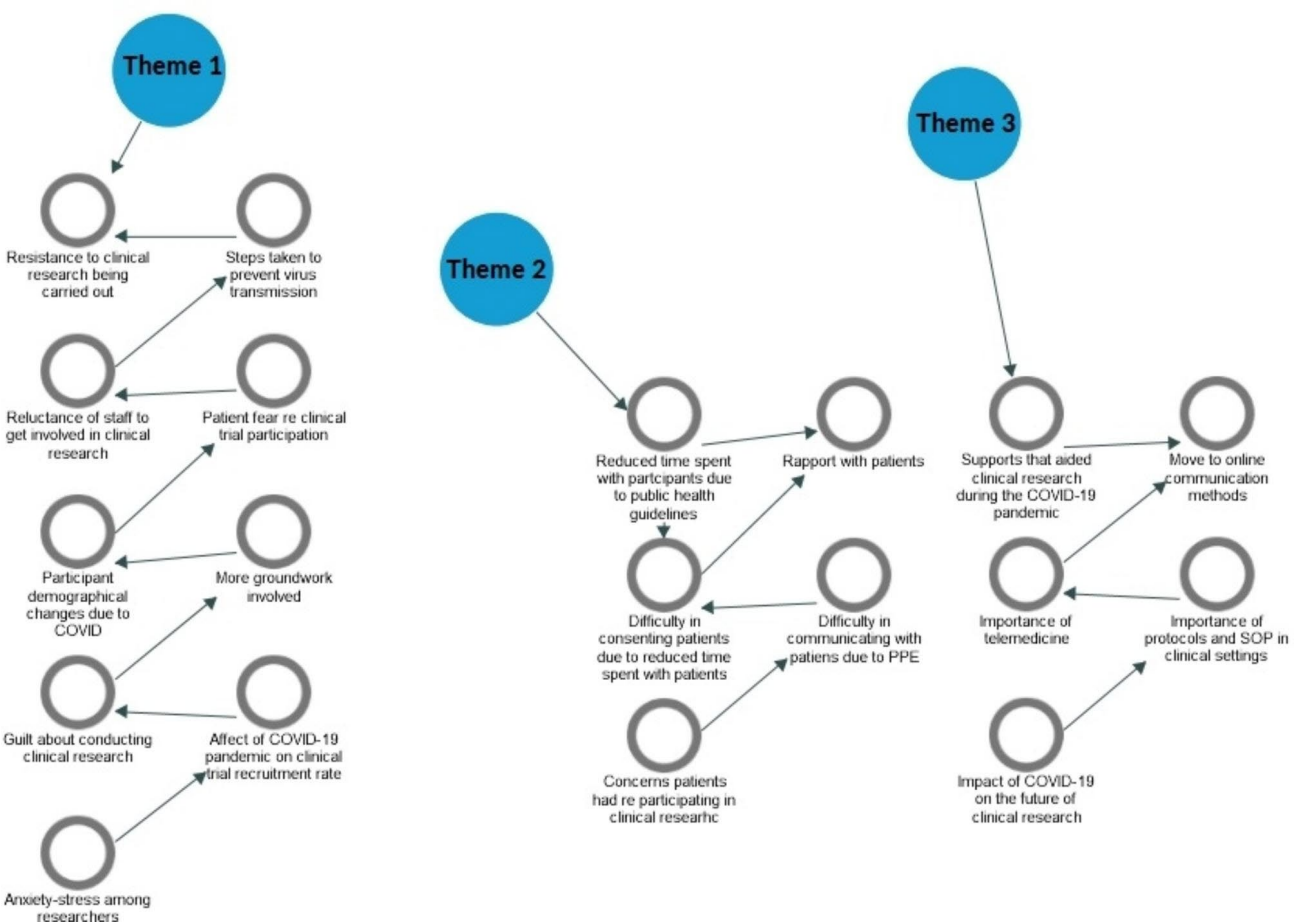
Theme Production. Overview of the production of themes resulting in three core themes; (1) The challenging COVID-19 clinical research landscape, (2) Clinical research communication barriers, and (3) Adaptations and learnings from clinical research during COVID-19

All interviews were audio recorded, fully transcribed, and reviewed by three researchers (AVW, LB and CF) to ensure accuracy. Transcribed interview data was analysed following a reflexive thematic approach [18]. The six steps of thematic analysis described by Braun and Clarke were adhered to, with particular emphasis on the flexible nature of this approach. The first step focused on data familiarisation which was achieved following transcription of interviews and reading of transcripts. The second step saw initial codes generated with the research question in mind. The third step was the identification of overlapping codes and preliminary themes. In the fourth step, themes were reviewed before being defined and named. Creation of a definition and narrative description for each theme, based around the research question led to the final selection of appropriate supporting data excerpts. Finally, the development of the manuscript with supporting quotes illustrated the themes identified. NVivo 12 was used to assist in data management and analysis with each transcript uploaded and the reflective thematic analysis steps outlined adhered to. Figure 1 provides an overview of theme production.

### Findings

Following data analysis, three principal themes were produced representing the experiences of CRS who were involved in clinical research during COVID-19: (1) The challenging COVID-19 clinical research landscape, (2) Clinical research communication barriers, and (3) Adaptations and learnings from clinical research during COVID-19 (Table 2).

**Table 2:** Summary of Themes

Theme	Sub-theme
Theme 1: The challenging COVID-19 clinical research landscape	1.a. COVID-19 exposure and transmission fears
	1.b. Presentation of a deteriorating patient cohort
	1.c. Research value
Theme 2: Clinical research communication barriers	2.a. Trust and rapport development
	2.b. Time implications
Theme 3: Clinical research adaptations and learnings	3.a. Adaptability, flexibility and accessibility of clinical research engagement
	3.b. Learning opportunities
	3.c. Sources of support

### Theme 1: the challenging COVID-19 clinical research landscape

#### 1.a. COVID-19 exposure and transmission fears

COVID-19 exposure and transmission concerns to both CRS and clinical research participants was found to be at the core of the clinical research landscape. Evidence of a heightened sense of uncertainty towards clinical research involvement posed for CRS was outlined:

*“So, there was a lot of additional concerns to consider. The first was research personnel. Some of these were worried about catching COVID, their personal health.” (P4).*

CRS highlighted apprehensive to take part in clinical research due to COVID-19 risks. Some patients were presenting to hospital to undergo procedures which had previously been cancelled and rescheduled, making clinical research participants reluctant to engage in clinical research, impacting on the recruitment:

*“What I did find with people I’ve been recruiting, a lot of them it’s their 2nd or 3rd time thinking they are having the surgery done, sometimes I find research isn’t their priority” (PT 2).*

CRS recognised challenges dealing with patients fears at having to be physically present in a clinical setting. These factors led to recruitment and retention issues, requiring reassurances for patients due to the deterrent to participation of the clinical environment:

*“Definitely they were very nervous about coming into the hospital and we had a lot of cancellations on follow up… Patients were nervous. They weren’t comfortable coming in at all. I think the last place they wanted to be was in a hospital*” (PT 1).

The relocation of acute hospital services as an infection control measure, coupled with the repurposing of clinical research areas to accommodate COVID-19 patients proved difficult for CRS, impacting recruitment and follow up phases:

*“So that [relocation of services] caused a problem in trying to do the research because we were doing 1 in a 24 h period… it was hard to keep track of where some of them were going” (P1).*

#### 1.b. Presentation of a deteriorating patient cohort

The demographic of patients presenting during COVID-19 had more complex needs requiring extensive medical care. This impacted CRS in terms of the amount of time needed to reassure patients, particularly impacting recruitment in the ED:

*“There was a marked change in how patients presented, it was hard to identify the patients… They were a lot sicker…, there was a significant decline in patients attending the ED from that time on” (P9).*

#### 1.c. Research value

A perceived lack of value on the role and need for clinical research during COVID-19 was evident. COVID-19 was seen to have had changed how HSCPs working in the clinical setting (HSCPs; including Nurses, Doctors, Occupational Therapists, Physiotherapists) appeared to view clinical research. This gap suggests some HSCPs did not recognise the contributions of clinical research or the need to conduct clinical research during COVID-19. This perception of clinical research appears to have been an underlying issue, challenges brought about during the pandemic appears to have exacerbated this issue:

*“Sometimes people might not see research as a priority…, but we know from our own background the importance of clinical research and though it may not be deemed as essential all the time” (PT8).*

Engagement challenges with HSCPs in clinical research processes during the pandemic highlighted tensions around the rationale for CRS with clinical roles to continue to utilise resources for clinical research activities during COVID-19:

*“… I was doing clinical research in the COVID-19 related area, and there was a lot of guilt because I know a lot of my colleagues thought ‘it’s disgraceful that you’re supporting research when we need staff who are intensive care trained” (P7).*

The varied sense of value towards continuing to conduct clinical research during COVID-19 created a negative impact for some CRS:

*“I don’t know was that a personal guilt… I really think we need to showcase the value of the research. I think we need to put our hats on the table - do we really value research?” (P7).*

### Theme 2: clinical research communication barriers

PPE presented significant communication challenges in the clinical research environment. As well as presenting difficulties in auditory aspects of communication, the broader rapport building element between CRS and patients was significantly affected:

*“Normally you sit beside the patient chatting while you complete paperwork, all non-essential examination activities had to take place two meters from the patient. It changed the patient’s relationship” (P4).*

PPE was found to negatively impact patients’ ability to hear and speak effectively with CRS, while hampering natural rapport development. This was further exacerbated for patients with hearing issues, particularly amongst older adult groups:

*“You were wearing a mask and you were gowned up as if you were in surgery, so it was harder to communicate and to build that rapport, and actually for people who are hard of hearing, the masks didn’t help matters” (P1).*

An area intensified by PPE communication issues was consent; anxiety amongst patients, as well as family not allowed to be in attendance resulted in challenges in the consent process:

*“It was a huge barrier because patients were a lot sicker… so it doubled your time recruiting, and if they did consent, I would follow up with family because they were in the ED and they had no family members with them to discuss it” (P9).*

### Theme 3: clinical research adaptations and learnings

#### 3.a. Adaptability, flexibility, and accessibility of clinical research engagement

Clinical research teams and broader HSCP teams showed innovation in adapting efforts to best manage COVID-19. Clinical research adaptations saw significant changes in communication, moving from in person to virtual communication which was positively viewed amongst participants:

“*Something positive is how adaptable we are. Across the hospital group, every department and ward, people showed tremendous tenacity” (P8).*

The change to online and phone communication enhanced some elements of the clinical research process, providing a more accessible and flexible means of engagement with patients as well as stakeholders:

*“We can do more over the phone, more telemedicine, more tele research, we don’t need to have patients coming into acute hospitals as much” (P7).*

However, this approach proved difficult for some CRS, largely due to IT challenges and availability of appropriate technology:

*“You had to adapt to this change, doing video and telephone calls, that brought its own barriers as people mightn’t have access to Wi-Fi or devices, how good they were at using devices, a lot of people don’t have devices” (P10).*

#### 3.b. Learning opportunities

Reflecting opportunities from increased online communication in clinical research, a need for enhanced training for stakeholders on IT issues was identified:

*“I think that [IT training] would be something we could have provided and provided information, that would be something that I’d definitely recommend” (P7).*

A common thread was to use learnings to inform protocols and guidelines to manage adverse events. Participants were keen for the development of operating procedures and protocols to minimise any impact on future clinical research:

*“We’re trying to plan for going forward, to incorporate a more virtual aspect to clinical research, there needs to be contingency plans in place, we need more ways of doing things… ways we can alter things to suit the situation” (P8).*

An outcome from the adaptations that emerged during COVID-19 was the need to consider the impact of the physical environment on clinical research. Participants spoke of fear and reservations from patients at the need to attend a clinical setting:

*“It made people think do we need to do things this way, does everything have to come into hospital or outpatients, can it take the pressure off and be done in a different location… people would be happier if they didn’t have to come in, sometimes it puts people off” (P10).*

#### 3.c. Sources of support

Participants highlighted key sources of support during COVID-19. The role of the infection control team was well recognised, providing CRS with a sense of reassurance in conducting their work safely:

*“Infection prevention and control were amazing, their presence was there, they went to all the wards, they demonstrated all the correct PPE use, that gave me huge confidence, it was a really fast and well-planned from them” (P8).*

Specific professional supports were acknowledged which were identified as providing clear and timely evidence-based guidance:

*“The Irish Research Nurses and Midwives’ Network were fantastic… I found they were most up to date and provided readily available information which I knew was most accurate at the time” (P7).*

The role of collegiality as a means of supporting clinical research during COVID-19 was widely acknowledged. Despite challenges faced, participants acknowledged the support from HSCPs and stakeholders in the clinical setting:

*“We pull together like people were so good, like, you know, people were so good, like they just pulled together to get the students through. They pull together to get the research through” (P3).*

## Discussion

This study found three principal themes which represented the experiences of CRS involved in clinical research during COVID-19: (1) The challenging COVID-19 clinical research landscape, (2) Clinical research communication barriers, and (3) Adaptations and learnings from clinical research during COVID-19.

In answering the study aim of exploring the experiences of conducting clinical research during COVID-19 with CRS, this study captured the range of factors that impacted conducting clinical research in the Irish context. Findings looked beyond operational elements to show how the altered clinical research landscape and profile of clinical research participants impacted clinical research. Additional clinical research challenges faced by CRS ranged from PPE communication barriers, a more unwell patient cohort, exposure concerns, infection control measures and stakeholder morale. Participants accounts of fear and anxiety experienced echoed frontline HSCPs in a US scoping review [9, 19].

While previous studies identified the telemedicine challenges in terms of reduced human interactions [20–22] findings from this study support a move towards telemedicine in offering greater flexibility and accessibility to CRS and participants. CRS emphasised a need for flexible communication approaches and patient engagement through consultations that occur off-site rather than acute hospitals. This study showed that participants valued a telemedicine approach for providing patients with greater control to engage in clinical research.

This study contributes to the future of clinical research in Ireland, providing pragmatic insights to inform protocols and procedures to manage future clinical research challenges. As reflected in the data, preparation for any future challenges like COVID-19 was seen as a priority amongst participants, with a focus on the need for protocol developments to minimise impacts to clinical research. In addition to protocol guidance, the need for communicating such guidance mirrored the importance of timely and relevant information from reputable sources [18, 23–25].

An area of particular importance that has not previously been identified in the literature is the clinical research value component. This study illustrated a sense of limited value placed on clinical research from some HSCPs, with clinical research not always seen as a priority during the pandemic. This raises issues around wider perceptions of clinical research amongst HSCPs, with participants expressing a lack of understanding on the need and relevance for clinical research, which appears to be seen as a separate entity to clinical practice. This area of research value and research perception must be explored to ensure stakeholders have a full sense of understanding and recognise the role clinical research has in enhancing patient care and outcomes.

This study addresses a gap in the literature by capturing the perspectives of CRS experiences of conducting clinical research during COVID-19. Findings build on an evidence base which has predominantly focused on the operational elements of clinical research during COVID-19, strengthening the evidence base to understand the clinical research landscape, communication recommendations relevant to CRS.

### Limitations

This study presented the first Irish research study to consider the impact of COVID-19 from clinical and academic stakeholders involved in clinical research activities. Given this study was conducted in one Hospital site in Ireland, there is scope to broaden nationally and internationally with comparison across different Hospital sites and participant experiences. As data was collected during a specific pandemic time point, capturing different timepoints would provide a more nuanced understanding of the wider set of experiences. Additionally, given challenges in data collection during COVID-19, a combination of both in-person and telephone interviews may have elicited extensive insights. Such limitations are useful to consider for future research undertaken in this area.

## Conclusion

This exploration of the experiences of CRS during COVID-19 provides insights into the challenges and complexities of conducting clinical research. This study highlights the impact that COVID-19 had in areas such as consent, recruitment, communication, rapport as well as strain amongst HSCPs in terms of the value and prioritisation of clinical research. Barriers and facilitators of clinical research adaptations towards virtual communication and the flexibility this offers a patient group with a preference for a non-clinical research setting provide learnings for the future clinical research.

The experience of CRS offers insight and recognition of the main challenges for clinical research during COVID-19, as well as unique insights into how CRS adapted to maintain clinical research activities. These experiences are central to understanding, supporting, and equipping CRS with necessary training, resources, and support to pre-empt any future disruption to clinical research as experienced during COVID-19.

### Electronic supplementary material

Below is the link to the electronic supplementary material.

Supplementary Material 1

## Data Availability

The datasets generated and/or analysed during the current study are not publicly available due to ongoing analysis of data but are available from the corresponding author on reasonable request.

## Abbreviations

HSCP: Health and Social Care Professionals
CRS: Clinical research stakeholders
