# Supplementary material for: Clinical research stakeholders’ experiences of clinical research during COVID-19: a qualitative study
Source: BMC Res Notes. 2023 Sep 30;16:240. doi: 10.1186/s13104-023-06534-5 (PMC10544112; doi:10.1186/s13104-023-06534-5)
Supplement: Supplementary file 1 — Supplementary Material 1 [file 13104_2023_6534_MOESM1_ESM.docx]

**Supplementary File 1: Interview Guide**

| **Topic area** | **Experience of participation in clinical research since the COVID-19 outbreak** | **Factors that support clinical research** | **Factors that impede clinical research** | **Future research** |
| --- | --- | --- | --- | --- |
| **Questions / prompts** | Can you tell me a little bit about your role in clinical research? | What have you found reassuring in your role since the outbreak? | What have you found challenging in your role since the outbreak? | How has COVID impacted on the role of clinical research into the future? |
|  | How has this role changed for you since the outbreak? | Is there any source of information that you have found reassuring?  Probe work, family, GP, helpline, article, news report or TV show | How have you overcome these challenges? | Probe:  Positive and negative impacts |
|  |  | Query supports related to concerns already reported |  |  |

Clinical research stakeholders’ experiences of clinical research during COVID-19: A qualitative study
